# Efficacy of a novel oral chewable tablet containing sarolaner, moxidectin and pyrantel (Simparica Trio™) against natural flea and tick infestations on dogs presented as veterinary patients in Europe

**DOI:** 10.1186/s13071-020-3946-1

**Published:** 2020-03-01

**Authors:** Csilla Becskei, Daphne Fias, Sean P. Mahabir, Robert Farkas

**Affiliations:** 1 Veterinary Medicine Research and Development, Zoetis, Mercuriusstraat 20, 1930 Zaventem, Belgium; 2 Veterinary Medicine Research and Development, Zoetis, 333 Portage St., Kalamazoo, MI 49007 USA; 3 Department of Parasitology and Zoology, University of Veterinary Medicine, István u. 2, Budapest, 1078 Hungary

**Keywords:** Dog, Efficacy, Fleas, Moxidectin, Oral, Sarolaner, Simparica Trio™, Pyrantel pamoate, Ticks, Veterinary patients

## Abstract

**Background:**

A novel chewable oral tablet containing sarolaner, moxidectin and pyrantel (Simparica Trio™) has recently been developed to provide persistent protection against flea and tick infections for a month, treatment of hookworm and roundworm infections and prevention of heartworm and lungworm disease in dogs. Two field studies were conducted to evaluate the safety and efficacy of Simparica Trio™ against natural flea and tick infestations on dogs in Europe.

**Methods:**

Dogs with natural flea or tick infestations were allocated randomly to treatment on Day 0 with either Simparica Trio™ tablets (flea study: *n* = 297; tick study: *n* = 189) to provide 1.2–2.4 mg/kg sarolaner, 24–48 µg/kg moxidectin and 5–10 mg/kg pyrantel (as pamoate salt) or with NexGard® Spectra (afoxolaner + milbemycin oxime) according to the label instructions (flea study: *n* = 164; tick study: *n* = 91). Efficacy was calculated based on the mean percent reduction in live parasite counts compared to the respective pre-treatment counts on Days 14 and 30 in the flea study and on Days 7, 14, 21 and 30 in the tick study. To count the fleas, the dog’s entire coat was systematically combed using an extra fine-tooth flea comb until all fleas were removed. For the tick counts, the dog’s entire coat was searched manually. Resolution of the clinical signs of flea allergy dermatitis (FAD) was assessed in flea allergic dogs in the flea study. Palatability was assessed in both studies.

**Results:**

Simparica Trio™ was well tolerated in both studies. Efficacy against fleas was ≥ 97.9% in the Simparica Trio™ group and ≥ 96.1% in the NexGard® Spectra group. Efficacy against ticks was ≥ 94.8% in the Simparica Trio™ group and ≥ 94.4% in the NexGard® Spectra group. Clinical signs of flea allergy dermatitis improved following treatment with Simparica Trio™. Simparica Trio™ tablets were voluntarily and fully consumed on ≥ 78% of the 485 occasions they were offered.

**Conclusions:**

A single oral dose of Simparica Trio™ was safe and highly efficacious against naturally occurring flea and tick infestations for 1 month on dogs. Clinical signs of FAD improved following treatment. Simparica Trio™ was voluntarily and readily consumed by most dogs.

## Background

Fleas are present throughout Europe and the cat flea (*Ctenocephalides felis*) is the species most commonly found on dogs, followed by the dog flea (*Ctenocephalides canis*) and the hedgehog flea (*Archaeopsylla erinacei*) [1]. Flea blood-feeding causes local irritation (flea bite dermatitis) and with repeated exposure dogs may develop flea allergy dermatitis [2, 3]. Heavy infestations can lead to anemia, especially in young or debilitated animals [2]. Fleas transmit several zoonotic disease agents including those that cause flea-borne spotted fever (*Rickettsia felis*), murine typhus (*Rickettsia typhi*) and cat scratch disease (*Bartonella henselae*) [4]. Fleas also serve as intermediate hosts for helminths such as the dog tapeworm *Dipylidium caninum*, also called the flea tapeworm [5].

Ticks are endemic throughout most of Europe and those from the genera *Dermacentor*, *Ixodes* and *Rhipicephalus* are commonly found infesting dogs [1]. Tick blood-feeding causes nuisance and skin irritation, and in heavy infestations can result in anemia [6]. Ticks transmit several disease-causing agents, some of which are zoonotic. *Dermacentor reticulatus* is known to be the vector of *Babesia canis canis*, and in Europe the causative agents of canine and human anaplasmosis (*Anaplasma phagocytophilum*) and Lyme borreliosis (*Borrelia burgdorferi* (*sensu lato*)) are transmitted by *Ixodes ricinus*. *Rhipicephalus* ticks are known to transmit the disease agents that cause canine ehrlichiosis (*Ehrlichia canis*), babesiosis (*Babesia vogeli*, *Babesia gibsoni*), hepatozoonosis (*Hepatozoon canis*) and Mediterranean spotted fever (*Rickettsia conorii*) [7].

Because of these above-mentioned health risks, it is recommended that dogs at risk of flea and tick infestation receive sustained flea and tick treatment with insecticidal and acaricide activity [1, 8]. To provide safe and efficacious treatment options for dogs to address this need of veterinarians and owners, there is a continuous need to develop new parasiticides, including combinations with various active ingredients. A chewable oral tablet that contains sarolaner, moxidectin and pyrantel has recently been approved (Simparica Trio™, Zoetis, Parsippany, NJ, USA) that provides treatment of flea and tick infestations for 1 month in dogs, as well as roundworm and hookworm infections, and prevention of heartworm and lungworm diseases. The two clinical field studies reported here were conducted to evaluate the palatability, safety, and efficacy of Simparica Trio™ against naturally occurring flea and tick infestations on dogs presented as veterinary patients in Europe.

## Methods

Two masked, randomized, positive-controlled field studies were conducted on dogs with natural flea or tick infestations, enrolled at 30 veterinary practices in Germany (10 sites), Hungary (10 sites), and Portugal (10 sites). Both studies were conducted in accordance with the World Association for the Advancement of Veterinary Parasitology (WAAVP) guidelines for evaluating the efficacy of parasiticides for the treatment, prevention and control of flea and tick infestation on dogs and cats [9] and complied with Good Clinical Practices [10]. Personnel involved in making assessments of efficacy or safety were masked to treatment assignments. Day 0 was defined as the day the treatment was administered.

### Animals

The patient population was recruited from dogs at least 8 weeks of age and at least 2.0 kg body weight. There were no breed or sex restrictions, however pregnant or lactating females or dogs intended for breeding were not eligible for enrollment. Dogs living in or with a history of travel to heartworm endemic regions were confirmed to be negative for heartworm (*Dirofilaria immitis*) antigen. Enrolled dogs had not received treatment with any ectoparasiticide with residual efficacy against fleas or ticks within 30 days of enrollment and had not received injectable moxidectin (Guardian®; Elanco, Greenfield, IN, USA) within the six months prior to study treatment.

Enrollment in both studies was restricted to households with a maximum of three dogs, and additionally in the flea study to households with a maximum of three cats. To be considered for enrollment, at least one dog in the household had to harbor ≥ 5 live adult fleas in the flea study or ≥ 3 live attached ticks in the tick study. In multiple dog households where more than one dog met the inclusion criteria, the investigator chose the first dog that harbored the minimum required number of fleas or ticks as the primary patient, and all other enrolled dogs were considered supplementary patients. Enrollment of all dogs in the household was required in the flea study but was optional at the discretion of the investigator or dog owner in the tick study. In both studies, efficacy assessments were conducted on primary patients, and palatability and safety assessments on both primary and supplementary patients.

### Flea and tick counts

Flea counts on primary patients were conducted pre-treatment on Day-1 or 0, and post-treatment on Days 14 (± 5) and 30 (± 5). The dog’s entire coat was systematically combed using an extra fine-tooth flea comb until all fleas were removed. Each dog was combed for a minimum of 10 min. If fleas were found in the last 5 min, then combing was continued in 5-min increments until no fleas were found in a 5-min period. All live fleas were counted as they were removed. Fleas capable of maintaining upright orientation and/or coordinated movement were considered alive.

Tick counts on primary patients were conducted pre-treatment on Day-1 or 0, and after treatment (on Day 0) on Days 7 (± 2), 14 (± 2), 21 (± 2) and 30 (± 4). The dog’s hair was manually pushed against its natural nap such that the skin and any attached ticks were exposed. The dog’s entire coat was thoroughly examined for at least 10 min. If ticks were found in the last 5 min, then the examination was continued in 5-min increments until no ticks were found in a 5-min period. All free and attached ticks were counted and removed, whether these were alive or dead. Ticks were considered attached if they had their mouth parts implanted into the animal, and live if they exhibited normal movement and behavior. Ticks that did not exhibit movement or response to external stimuli were considered dead.

Collected fleas and ticks were preserved in isopropyl alcohol and sent to a central parasitology laboratory (University of Veterinary Medicine, Department of Parasitology and Zoology, Budapest, Hungary) for determination of the species and sex of the fleas, and the species, developmental stage and sex of adult ticks [11, 12].

### Clinical signs of flea allergy dermatitis (FAD)

In the flea study, each primary patient was examined by a veterinarian to assess the dog for clinical signs of flea infestation on the same days when flea counts were performed (pre-treatment and 14 and 30 days after treatment). The severity of pruritus, erythema, scaling, alopecia, and dermatitis/pyodermatitis attributed by the veterinarian to flea infestation were graded as absent (no observable abnormalities), mild (the intensity/density of the abnormality was low and only a small area of the dog’s body was affected), moderate (the abnormality was of great intensity/density over a small area or was of lesser intensity/density but affected a large area of the dog’s body) or severe (the abnormality was of great intensity/density and covered a large area of the dog’s body). At study completion on Day 30, the veterinarian identified dogs suspected of having FAD based on review of the dog’s history and clinical signs of flea infestation during the study.

### Randomization

Primary dogs were allocated to one of the two treatments according to a pre-determined generalized randomized block design with a one-way treatment structure replicated in multiple clinics. At each clinic, dogs were blocked based on order of enrolment. Dogs were blocked in groups of three and allocated randomly to treatment with sarolaner + moxidectin + pyrantel (Simparica Trio™) or afoxolaner + milbemycin oxime (NexGard® Spectra; Boehringer-Ingelheim, Ingelheim, Germany) in a 2:1 ratio. Any supplementary dogs received the same treatment as the primary dog in the same household.

### Treatment and palatability assessment

Simparica Trio™ was provided in six different tablet strengths to provide dose ranges of 1.2–2.4 mg/kg sarolaner, 24–48 µg/kg moxidectin and 5–10 mg/kg pyrantel (as pamoate salt). NexGard® Spectra (afoxolaner + milbemycin oxime) was dosed according to the commercial product label instructions.

Treatments were administered on Day 0 by the dog owner in the home environment. Dose was determined based on the body weight recorded on Day-1 or 0. There were no restrictions regarding the prandial state at the time of treatment administration, therefore, tablets could be administered at any time regardless of the dog’s normal feeding schedule.

In order to assess tablet palatability, the tablet(s) were offered either in an empty food bowl or by hand and the dog was given 2 min to consume the entire dose. If the entire dose was not consumed within 2 min, then the remaining whole or partial tablets were offered with a small quantity of food or administered directly by ‘pilling’ in order to ensure complete dosing. Following each dosing, the dogs were observed for several minutes for immediate adverse reactions.

### Safety assessments

All abnormal health events observed by the veterinarian during physical examinations or observed by the owner were recorded, as were any concomitantly administered medications. Physical examinations that also included the measurement of rectal temperatures were performed by a suitably trained veterinarian on all primary and supplementary dogs prior to treatment administration. Post-treatment, physical examinations were performed on primary dogs in the flea study on Days 14 (± 5) and 30 (± 5), and in the tick study on Days 7 (± 2), 14 (± 2), 21 (± 2) and 30 (± 4). Physical examinations on supplementary dogs were mandatory in both studies at the final visit on Day 30 (flea study, ± 5 days; tick study, ± 4 days).

### Data analysis

All dogs that received treatment were included in the safety assessments. Only primary dogs were included in the efficacy analysis. The primary dog in each household was the experimental unit and the primary endpoint was the live flea or tick count.

Efficacy was calculated for each post-treatment day as the percentage reduction in live parasite counts compared to the pre-treatment counts for each animal using the following formula: [(C − T)/C] × 100, where C is the pre-treatment live parasite count and T is the post-treatment live parasite count.

Percent efficacy was analyzed using a general linear mixed model for repeated measures (SAS version 9.4). The model included the fixed effects of treatment, time-point, and treatment by time-point interaction and the random effects of study site, interaction between study site and treatment, block within study site, animal within block, study site and treatment, the interaction of study site, treatment and time-point, and error. Least squares means were used as estimates of the treatment means at each time-point. Standard errors for the least squares means were calculated and 95% confidence intervals were constructed by time-point.

Non-inferiority across all flea species and tick species was evaluated at each post-treatment time-point using a margin of 15% at the one-sided 0.025 significance level. In effect this meant that if the lower 97.5% confidence limit of the treatment difference (Simparica Trio™–NexGard® Spectra) was greater than − 15% then Simparica Trio™ was declared non-inferior at that time-point.

Improvement in the clinical signs of FAD was assessed on primary dogs that were identified by the veterinarians as having flea allergy dermatitis. Tablet palatability was assessed by calculating the percentage of treatment administrations in which the entire prescribed dose was voluntarily consumed within two minutes.

## Results

### Patient signalment

Patient signalment is summarized in Table 1.

**Table 1:** Signalment of primary dogs enrolled as veterinary patients in studies conducted in Europe to evaluate the efficacy of Simparica Trio™ against natural flea and tick infestations

Signalment	Flea study		Tick study	
	Simparica Trio™ (*n* = 180)	Afoxolaner + Milbemycin oxime (*n* = 98)	Simparica Trio™ (*n* = 126)	Afoxolaner + Milbemycin oxime (*n* = 64)
Purebred	64	41	71	41
Non-purebred	116	57	55	23
Age, mean (years)	4.8	4.3	4.7	4.7
Age, range (years)	0.2–15.0	0.2–16.0	0.2–14.0	0.3–14.0
Body weight, mean (kg)	14.2	15.5	19.4	21.1
Body weight, range (kg)	2.1–48.8	2.7–50.9	2.1–65.1	2.7–54.3
Male	98	52	75	38
Female	82	46	51	26
Lives mostly indoors	20	7	11	4
Lives mostly outdoors	116	63	73	45
Lives indoors and outdoors	44	28	42	15

#### Flea study

Two hundred seventy-eight primary dogs (180 Simparica Trio™ and 98 afoxolaner + milbemycin oxime) and 183 supplementary dogs (117 Simparica Trio™ and 66 afoxolaner + milbemycin oxime) were enrolled and treated.

Three Simparica Trio™-treated primary dogs and one afoxolaner + milbemycin-treated primary dog did not complete the study. In the Simparica Trio™ group one primary dog died in a road traffic accident, one was withdrawn by the owner without providing a reason, and one was withdrawn due to an unrelated medical condition (pyelonephritis). In the afoxolaner + milbemycin group, one primary dog was withdrawn due to a randomization error. Loss of any primary dog resulted in the automatic removal of any supplementary dogs from the same household which resulted in the withdrawal of one supplementary dog from each treatment group.

#### Tick study

One hundred ninety primary dogs (126 Simparica Trio™ and 64 afoxolaner + milbemycin oxime) and 90 supplementary dogs (63 Simparica Trio™ and 27 afoxolaner + milbemycin oxime) were enrolled and treated. All dogs completed the entire study except for one Simparica Trio™-treated primary dog that died in a vehicular accident.

### Palatability assessment

Both treatments were well accepted and the palatability of Simparica Trio™ and NexGard® Spectra was nearly identical in both studies. In the flea study, 77.7% of the 296 Simparica Trio™ doses and 79.1% of the 163 afoxolaner + milbemycin oxime doses were voluntarily consumed within 2 min of offering without food/treats. In the tick study, 79.9% of the 189 Simparica Trio™ doses and 79.1% of the 91 afoxolaner + milbemycin oxime doses were voluntarily consumed within 2 min of offering without food.

### Safety

There were no treatment-related abnormal health events in any of the studies. The overall incidence of adverse events was low, with post-treatment abnormal health reported in ≤ 3.2% of dogs in either study. The frequency and types of events were consistent with those expected to occur in a general population of dogs infested with ectoparasites and included conjunctivitis, pyelonephritis, otitis externa, diarrhoea, tick granulomas, weakness and apathy in the Simparica Trio™ groups and diarrhoea and tick granuloma in the control groups. The death of two Simparica Trio™-treated dogs due to vehicular accidents were the only serious adverse events in either study.

Forty-five (9.3%) Simparica Trio ™-treated dogs received a variety of concomitant medications, including vaccines, anti-inflammatory medications, vitamins, antacids, antiprotozoal agents, and ophthalmic and otic medications. In both studies, Simparica™ Trio was well tolerated when administered alone or concomitantly with a variety of other medications commonly used in veterinary medicine.

### Efficacy

#### Flea study

The results for all flea species combined are summarized in Table 2. Pre-treatment arithmetic mean live flea counts were 12.2 (range 5–111) in the Simparica Trio™ group and 10.4 (range 5–59) in the afoxolaner + milbemycin oxime group. Compared to pre-treatment, on Days 14 and 30 mean live flea counts were reduced by 97.9% and 98.6%, respectively, in the Simparica Trio™ group, and by 96.1% and 98.7%, respectively, in the afoxolaner + milbemycin oxime group. The efficacy of Simparica Trio™ was non-inferior to that of NexGard® Spectra on Days 14 (*t*_(59)_ = 8.66, *P* < 0.0001, 95% CI: − 2.2–5.5) and 30 (*t*_(19.2)_ = 11.71, *P* < 0.0001, 95% CI: − 2.4–3.1).

**Table 2:** Efficacy of a single oral dose of Simparica Trio™ and afoxolaner+ milbemycin oxime against natural flea infestations across all flea species on dogs presented as veterinary patients in Europe

Study day	Treatment group	*n*	Live flea counts		% Efficacy
			Range	Arithmetic mean	
− 1 to 0	Simparica Trio™	176	5–111	12.2	–
	Afoxolaner + Milbemycin oxime	95	5–59	10.4	–
14 ± 5	Simparica Trio™	174	0–11	0.2	97.9
	Afoxolaner + Milbemycin oxime	91	0–12	0.5	96.1
30 ± 5	Simparica Trio™	170	0–17	0.2	98.6
	Afoxolaner + Milbemycin oxime	91	0–4	0.2	98.7

The number and proportions of dogs infested with each flea species and the mean flea counts by study day for each species are summarized in Table 3. At enrolment, 79.3% of dogs were infested with *C. felis* (cat flea), 18.5% with *C. canis* (dog flea), 18.5% with *Pulex irritans* (human flea) and 1.9% with *A. erinacei* (hedgehog flea). Against each of these individual flea species, by Day 30 Simparica Trio™ reduced live flea counts by ≥ 98.9% and afoxolaner + milbemycin oxime by ≥ 98.1%.

**Table 3:** Efficacy of a single oral dose of Simparica Trio™ and afoxolaner+ milbemycin oxime against natural infestations with *Ctenocephalides felis*, *Ctenocephalides canis*, *Pulex irritans* and *Archaeopsylla erinacei* on dogs presented as veterinary patients in Europe

Treatment group	Study day	*C. felis*		*C. canis*		*P. irritans*		*A. erinacei*	
		Mean flea count	% Efficacy	Mean flea count	% Efficacy	Mean flea count	% Efficacy	Mean flea count	% Efficacy
Simparica Trio™	− 1 or 0	12.4		8.9		4.4		2.0	
	14 ± 5	0.3	97.6	0	100	0	100	0	100
	30 ± 5	0.2	98.9	0	100	0	99.7	0	100
Afoxolaner + Milbemycin oxime	− 1 or 0	9.6		7.2		6.1		6.0	
	14 ± 5	0.5	96.2	0	100	0.2	97.8	1.3	89.7
	30 ± 5	0.2	98.1	0	100	0	100	0	100.0

Note: Before treatment administration (on study day − 1 or 0) the number of dogs in the Simparica Trio™ and afoxolaner + milbemycin oxime groups infected with each species was 139 and 76 for *C. felis*, 33 and 17 for *C. canis*, 34 and 16 for *P. irritans *and 2 and 3 for *A. erinacei*, respectively

Based on medical history and improvement in clinical signs after treatment, 10 dogs in the Simparica Trio™ group and nine dogs in the afoxolaner + milbemycin oxime group were identified as having FAD. Prior to treatment, Simparica Trio™ dogs had clinical signs of pruritus (100%), erythema (60%), scaling (70%), alopecia from self-trauma (60%) and dermatitis/pyodermatitis (30%) (Table 4). On Day 30, these clinical signs improved, with Simparica Trio™-treated dogs showing lower prevalence of pruritus (20%), erythema (10%), scaling (10%), alopecia from self-trauma (0%) and dermatitis/pyodermatitis (10%). Prior to treatment with afoxolaner + milbemycin oxime, dogs had clinical signs of pruritus (100%), erythema (88.9%), scaling (88.9%), alopecia from self-trauma (55.6%) and dermatitis/pyodermatitis (55.6%). By Day 30, the prevalence of pruritus (12.5%), erythema (0%), scaling (25%), alopecia from self-trauma (0%) and dermatitis/pyodermatitis (0%) had improved.

**Table 4:** Efficacy of a single oral dose of Simparica Trio™ and afoxolaner+ milbemycin oxime against natural flea infestations on dogs presented as veterinary patients in Europe: percentage of flea allergic dermatitis dogs with clinical signs

Treatment group	Assessment day	*n*	Pruritus	Erythema	Scaling	Alopecia from self-trauma	Dermatitis/Pyodermatitis
Simparica Trio™	− 1 or 0	10	100	60.0	70.0	60.0	30.0
	14 ± 5	10	30.0	10.0	30.0	10.0	10.0
	30 ± 5	10	20.0	10.0	10.0	0	10.0
Afoxolaner + Milbemycin oxime	− 1 or 0	9	100	88.9	88.9	55.6	55.6
	14 ± 5	8	37.5	12.5	50.0	37.5	12.5
	30 ± 5	8	12.5	0	25.0	0	0

#### Tick study

The results for all tick species combined are summarized in Table 5. Pre-treatment arithmetic mean live tick counts were 8.4 (range 3–191) in the Simparica Trio™ group and 9.0 (range 3–233) in the afoxolaner + milbemycin oxime group. Compared to pre-treatment, on Days 7, 21, 14 and 30 mean live tick counts were reduced by 96.6%, 94.8%, 98.1% and 98.6%, respectively, in the Simparica Trio™ group, and by 94.4%, 97.6%, 97.8% and 99.4%, respectively, in the afoxolaner + milbemycin oxime group. The efficacy of Simparica Trio™ was non-inferior to that of afoxolaner + milbemycin oxime on all days (Day 7: *t*_(189)_ = 4.35, *P* < 0.0001, 95% CI: − 5.3–10.8); Day 14: *t*_(171)_ = 3.77, *P* = 0.0009, 95% CI: − 10.6–4.1; Day 21: *t*_(70.2)_ = 7.77, *P* < 0.0001, 95% CI: − 3.2–4.9; Day 30: *t*_(49.4)_ = 7.36, *P* < 0.0001, 95% CI: − 4.6, 3.2).

**Table 5:** Efficacy of a single oral dose of Simparica Trio™ and afoxolaner+ milbemycin oxime against natural tick infestations across all species on dogs presented as veterinary patients in Europe

Study day	Treatment group	*n*	Live tick counts		% Efficacy
			Range	Arithmetic mean	
− 1 to 0	Simparica Trio™	126	3–191	8.4	–
	Afoxolaner + Milbemycin oxime	64	3–233	9.0	–
7 ± 2	Simparica Trio™	126	0–29	0.5	96.6
	Afoxolaner + Milbemycin oxime	64	0–13	0.6	94.4
14 ± 2	Simparica Trio™	126	0–15	0.4	94.8
	Afoxolaner + Milbemycin oxime	64	0–10	0.3	97.6
21 ± 2	Simparica Trio™	126	0–5	0.1	98.1
	Afoxolaner + Milbemycin oxime	64	0–2	0.1	97.8
30 ± 4	Simparica Trio™	126	0–4	0.1	98.6
	Afoxolaner + Milbemycin oxime	64	0–8	0.2	99.4

The number and proportions of dogs infested with each tick species and the mean tick counts by study day for each species are summarized in Table 6. At enrolment, 55.8% of dogs were infested with *I. ricinus* (castor bean tick), 47.4% with *Rhipicephalus sanguineus* (brown dog tick), 23.2% with *D. reticulatus* (ornate dog tick), and 2.6% with *Ixodes hexagonus* (hedgehog tick). Against each of these individual tick species, by Day 30 Simparica Trio™ reduced live tick counts by ≥ 97.0% and afoxolaner + milbemycin oxime by ≥ 98.1%.

**Table 6:** Efficacy of a single oral dose of Simparica Trio™ and afoxolaner+ milbemycin oxime against natural infestations with *Ixodes ricinus*, *Rhipicephalus sanguineus*, *Dermacentor reticulatus* and *Ixodes hexagonus* on dogs presented as veterinary patients in Europe

Treatment group	Study day	*I. ricinus*		*R. sanguineus*		*D. reticulatus*		*I. hexagonus*	
		Mean tick count	% Efficacy	Mean tick count	% Efficacy	Mean tick count	% Efficacy	Mean tick count	% Efficacy
Simparica Trio™	− 1 or 0	3.5	–	13.7	–	1.8	–	1.0	–
	7 ± 2	0.1	99.1	1.1	96.6	0	100	0	100
	14 ± 2	0.1	98.8	0.8	93.9	0	100	0	100
	21 ± 2	0.1	99.1	0.2	97.2	0	100	0	100
	30 ± 4	0	100.0	0.2	97.0	0	100	0	100
Afoxolaner + Milbemycin oxime	− 1 or 0	3.3	–	15.3	–	2.0	–	2.7	–
	7 ± 2	0.3	95.2	0.9	97.8	0	100	0	100
	14 ± 2	0.1	96.9	0.6	97.9	0	100	0	100
	21 ± 2	0.1	96.4	0.1	99.3	0	100	0	100
	30 ± 4	0	99.5	0.3	99.1	0	100	0	100

*Notes*: Before treatment administration (on study day − 1 or 0) the number of dogs in the Simparica Trio™ and afoxolaner + milbemycin oxime groups infected with each species was 71 and 35 for *I. ricinus*, 60 and 30 for *R. sanguineus*, 28 and 16 for *D. reticulatus* and 3 and 3 for *I. hexagonus*, respectively

## Discussion

In these studies, a single oral dose of Simparica Trio™ was safe and highly effective against natural flea and tick infestations on dogs under field conditions where they are continuously exposed to environmental re-infestation.

In the flea study, live flea counts for all species combined were reduced by 97.9% within two weeks after treatment, and this efficacy persisted through the end of the recommended monthly treatment interval with 98.6% reduction 30 days after treatment. These results are comparable to the efficacy reported previously for other orally administered pulicidal medications belonging to both the isoxazoline [13–15] and spinosyn class [16] in dogs. This high level of flea control is consistent with the adulticidal activity and breaking of the flea life-cycle through the cessation of flea reproduction that was seen in Simparica Trio™ laboratory efficacy studies [17]. Against induced infections with adult *C. felis* and *C. canis*, a single oral dose of Simparica Trio™ reduced live flea counts by ≥ 99.9% within 24 hours of treatment and by 100% within 24 hours of weekly re-infestation for 35 days [17]. Blood-feeding for at least 24 hours is required for a female flea to commence egg laying, thus fleas killed within 24 hours are not able to contribute to environmental contamination by flea eggs [18, 19]. The impact of rapid kill effect against adult fleas provided by Simparica Trio™ on flea egg-laying was confirmed in a study in which no flea eggs were recovered from any Simparica Trio™-treated dog during 20-hour egg collection periods that followed weekly infestations with adult *C. felis* for 35 days after treatment [17].

A rapid reduction in the number of adult fleas also contributes to the management of FAD [3], the most common veterinary dermatologic condition in the world [20]. This effect was observed in the present study where there was a substantial reduction in the clinical signs of FAD in dogs following treatment with Simparica Trio™. Pruritus is the predominant clinical sign in flea allergic dogs [21] and is the sign most often observed by dog owners [20]. In the present study, 100% of the flea allergic dogs treated with Simparica Trio™ had clinical signs of pruritus prior to treatment, and this was reduced to 30% of dogs at 14 days after treatment and to 20% of dogs at 30 days after treatment. Similar results were observed in a larger population of flea allergic dogs treated with Simparica Trio™ in a study conducted to evaluate efficacy against fleas on dogs in the USA [22]. In the USA study, 45.7% (58 of 127) of flea-infested dogs treated with Simparica Trio™ had clinical signs of pruritus prior to treatment, and this was reduced to 19% of dogs within 30 days after the first treatment and to only 6.9% of dogs within 30 days after the second monthly treatment.

In the tick study, live tick counts for all species combined were reduced by 96.6% within one week after treatment, and this efficacy persisted through the end of the recommended monthly treatment interval with 98.6% reduction 30 days after treatment. Similar studies evaluating oral parasiticides in dogs have demonstrated comparable efficacy against ticks in Europe [13, 14, 23]. The results of the present field studies are consistent with laboratory studies that confirmed the efficacy of a single oral dose of Simparica Trio™ against four tick species commonly infesting dogs in Europe. Against existing infestations of *D. reticulatus*, *I. hexagonus*, *I. ricinus* and *R. sanguineus*, Simparica Trio™ reduced live tick counts by ≥ 98.9% at 48 hours after treatment. Against weekly re-infestations, live *D. reticulatus* counts were reduced by ≥ 97.2% at 48 hours after infestation for 28 days, and live *I. hexagonus*, *I. ricinus* and *R. sanguineus* counts were reduced by ≥ 97.2% for 35 days [24].

The field study presented here confirms that Simparica Trio™ provides sustained tick control throughout the recommended month-long treatment interval for dogs that may be continuously exposed to environmental re-infestation. Sustained tick control is essential not only to reduce the direct effects of tick infestation, but also to reduce the potential for transmission of tick-borne pathogens [1, 25]. The laboratory studies demonstrated that Simparica Trio™ is highly effective against the four tick species that commonly infest dogs in Europe within 48 hours of treatment of an existing infestation and within 48 hours of re-infestation for one month following treatment. Tick species, host, climate, and disease agent properties all contribute to variability in the time between tick attachment for feeding and transmission of disease-causing agents [26]; however, removing or killing ticks within 48 hours of attachment should reduce the potential risk for the transmission of tick-borne pathogens e.g. *Babesia* spp. [27, 28].

Simparica Trio™ was well tolerated by the dogs. The observed few abnormal health events during the study were mild and transient in nature and are not unexpected in the general dog population.

The excellent efficacy against fleas and ticks observed in these studies, combined with efficacy for the prevention of heartworm and lungworm disease [29, 30], and the treatment of roundworm and hookworm infections [31, 32] in a single palatable tablet should make Simparica Trio™ a convenient option for veterinarians and pet owners to routinely treat and control many of the common external and internal parasites of dogs.

## Conclusions

Simparica Trio™ administered orally to provide dose ranges of 1.2–2.4 mg/kg sarolaner, 24–48 µg/kg moxidectin and 5–10 mg/kg pyrantel (as pamoate salt) was safe and highly effective against natural flea and tick infestations on dogs presented as veterinary patients. The rapid elimination of fleas provided by Simparica Trio™ resulted in the improvement of the clinical signs of flea allergy dermatitis in flea allergic dogs. Simparica Trio™ tablets were voluntarily and fully consumed by most dogs, thus providing a convenient option for the treatment and control of flea and tick infestations on dogs for an entire month.

## Data Availability

All data generated or analysed during this study are provided within the article.

## Abbreviations

FAD: flea allergy dermatitis
WAAVP: World Association for the Advancement of Veterinary Parasitology
